# Identification of VP1 peptides diagnostic of encephalomyocarditis virus from swine

**DOI:** 10.1186/s12985-014-0226-8

**Published:** 2014-12-30

**Authors:** Juan Bai, Xinhui Chen, Kangfu Jiang, Basit Zeshan, Ping Jiang

**Affiliations:** Key Laboratory of Animal Diseases Diagnostic and Immunology, Ministry of Agriculture, College of Veterinary Medicine, Nanjing Agricultural University, Nanjing, 210095 China; Jiangsu Co-innovation Center for Prevention and Control of Important Animal Infectious Diseases and Zoonoses, Yangzhou, China

**Keywords:** Encephalomyocarditis virus (EMCV), VP1, McAbs, Epitopes

## Abstract

**Background:**

Encephalomyocarditis virus (EMCV) can cause myocarditis, respiratory failure, reproductive failure, and sudden death in pre-weaned piglets, which has been isolated in China. EMCV VP1 protein was one of the most important structural proteins and played an important role in the protective immunity. In this study, 10 monoclonal antibodies (McAbs) against EMCV VP1 were screened and identified.

**Results:**

Epitope mapping results indicated that McAbs (6E11, 7A7, 7C9) specifically recognized the linear epitopes V(2)ENAEK(7), McAbs (1D1, 2A2, 5A1, 5A11, 5G1) recognized the epitope F(19)VAQPVY(25), and McAbs 1G8 and 3A9 recognized P(42)IGAFTVK(49). Protein sequence alignment of VP1 with 16 EMCV isolates indicated that the epitope F(19)VAQPVY(25) was conserved in all the reference strains. The epitopes P(42)IGAFTVK(49) and V(2)ENAEK(7) only had 1 or 2 variable amino acid among the reference strains. The 3D model analysis results showed that these epitopes presented as spheres were shown within the context of the complete particle.

**Conclusions:**

In this study, ten McAbs against EMCV VP1 were developed and three B-cells epitopes (2-7aa, 19-25aa and 42-49aa) were defined in VP1. All the results herein will promote the future investigations into the function of VP1 of EMCV and development of diagnostic methods of EMCV.

## Background

Encephalomyocarditis virus (EMCV) is characterized by not only myocarditis and encephalitis, but also neurological diseases, reproductive disorders and diabetes in many mammalian species [1-8]. Rodents are considered to be natural hosts of EMCV and are thought to be the primary reservoir and disseminators of the virus. Out of all domestic animals, pigs are the most susceptible to EMCV infection, which can cause severe economic losses on pig production due to high mortality in piglets as a result of respiratory failure [2,9,10] and in sows as a result of myocarditis and reproductive failure [11-13].

EMCV is a member of the *Cardiovirus* genus of *Picornaviridae* [14] and has a single-stranded positive-sense RNA of approximately 7.8 kb [15]. The ORF encodes for a polyprotein that comprises both non-structural and structural elements divided into three primary precursor molecules, namely P1, P2 and P3, encoding for 11 distinct proteins. The structural proteins VP4, VP2, VP3 and VP1 make up the viral capsid and are encoded in the P1 region towards the 5′-end of the genome. The viral capsid proteins, in their capacity to interact with cellular receptors, are crucial for this entry step and may be considered to be factors that can modulate EMCV virulence [1]. The three major capsid proteins, VP1, VP2 and VP3 that constitute the external virion shell of picornaviruses, are considered to play a pivotal role in viral infection and host recognition [16]. Among them, VP1 is one of the most antigenic and can stimulate the organism to produce neutralization antibody [17,18].

The detailed analysis of epitopes is important for the understanding of immunological events and for the development of epitope-based marker vaccines and diagnostic tools for various diseases [19-21]. In this paper, VP1 protein of EMCV NJ08 strain was expressed by the *Escherichia coli* system and ten monoclonal antibodies (McAbs) against the recombinant EMCV VP1 were screened and identified. Three linear epitopes (2-7aa, 19-25aa and 42-49aa) were identified in VP1 protein of EMCV.

## Results

### Development of monoclonal antibodies against VP1 protein

**Figure 1:**
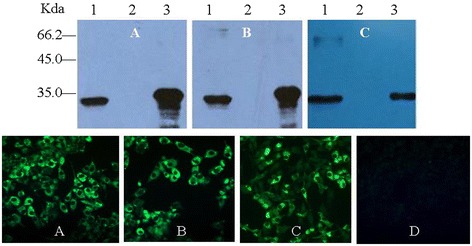
**Identification of McAbs with Western blot (up) and IFA (down). A**. 1D1; **B**. 2A2; **C**. 6B11; **D**. 1G8. Western blot: Lane1. purified EMCV; Lane2. BHK21 cells lyses; Lane3. recombinant VP1 expressed in *E. coli* BL21 containing pET-28a-VP1 plasmid.

After screening of the fusion cells by indirect ELISA, the positive hybridoma cells secreting the antibodies against VP1 protein were selected and sub-cloned thrice by limiting dilution, and ten McAbs were generated and named as 1D1, 1 F3, 1G8, 1D1, 2A2, 5A1, 5A11, 5G1, 6E11, 7A7 and 7C9. The results of Western blot showed that these McAbs were all directed against the purified EMCV and rVP1 protein expressed in *E. Coli* (Figure 1). Meanwhile, IFA results also showed that these McAbs could react with the EMCV in BHK21 cells (Figure 1). The titres of antibodies in the cells cultures were 1:1600–3200.

### Expression and identification of the truncated fragments

**Figure 2:**
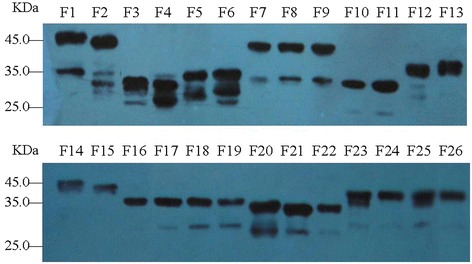
**The truncated fragments F1 ~ F26 were identified by Western blot with GST-tagged monoclonal antibody.**

Twenty-six overlapping VP1 protein gene fragments were prepared by PCR and cloned into a GST fusion protein expression vector. After validation by restriction analysis and nucleotide sequencing, recombinant proteins encoded by each of these constructs were expressed in *E.coli*. The resulting recombinant proteins were identified using Western blot with GST-tagged monoclonal antibody. The results showed that all the proteins were reactive with GST-tagged McAb (Figure 2).

### Mapping of the epitopes in VP1

**Figure 3:**
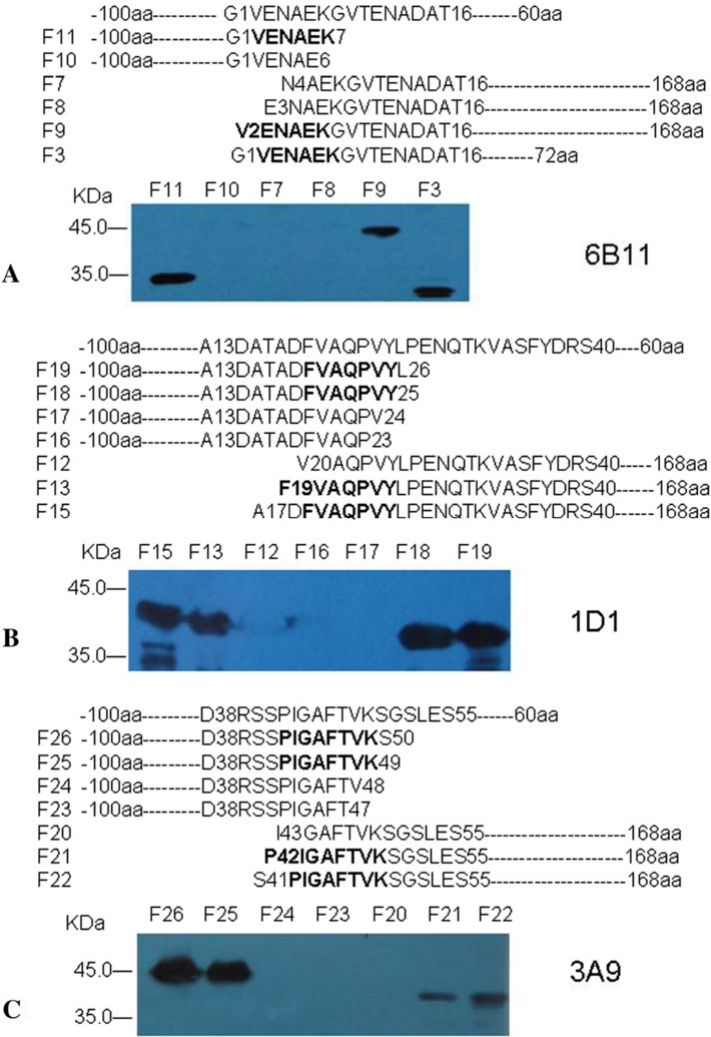
**Epitope mapping of McAbs by Western blot. The truncated fragments were detected with McAbs. (A)** McAb 6B11 specifically reacted with proteins which containing V(2)ENAEK(7) sequence; McAbs 7A7 and 7C9 have the same characteristic as 6E11(data not shown); **(B)** McAb 1D1 specifically reacted with proteins which containing F(19)VAQPVY(25) sequence; McAbs 2A2, 4B9, 4 F1, 5A1, 5A11 and 5G1 have the same characteristic as 1D1(data not shown); **(C)** McAb 3A9 specifically reacted with proteins which containing P(42)IGAFTVK(49) sequence; McAb 1G8 has the same characteristic as 3A9(data not shown).

In order to map the minimal sequences of the epitopes recognized by McAbs, the series of truncated recombinant proteins were used in Western blot was to identify the reactivity of each of 10 anti-VP1 McAbs. The results showed that McAbs (6E11, 7A7 and 7C9) could reacted with fragments (F11, F9 and F3) containing the six amino acids V(2)ENAEK(7), but not reacted with the fragments with the deletion of any one of the six amino acids (F10, F7 and F8). It indicated that amino acids V(2)ENAEK(7) in VP1 were essential for this epitope (Figure 3A). In the same way, McAbs (1D1, 2A2, 5A1, 5A11 and 5G1) reacted against the epitope comprising F(19)VAQPVY(25), because they could react with the fragments F15, F13, F18 and F19, but not F12, F16 and F17 (Figure 3B). McAbs (1G8 and 3A9) could reacted with fragments (F25, F26, F21 and F22) containing the eight amino acids P(42)IGAFTVK(49), but not reacted with the fragments with the deletion of any one of the eight amino acids (F24, F23 and F20) (Figure 3C).

**Figure 4:**
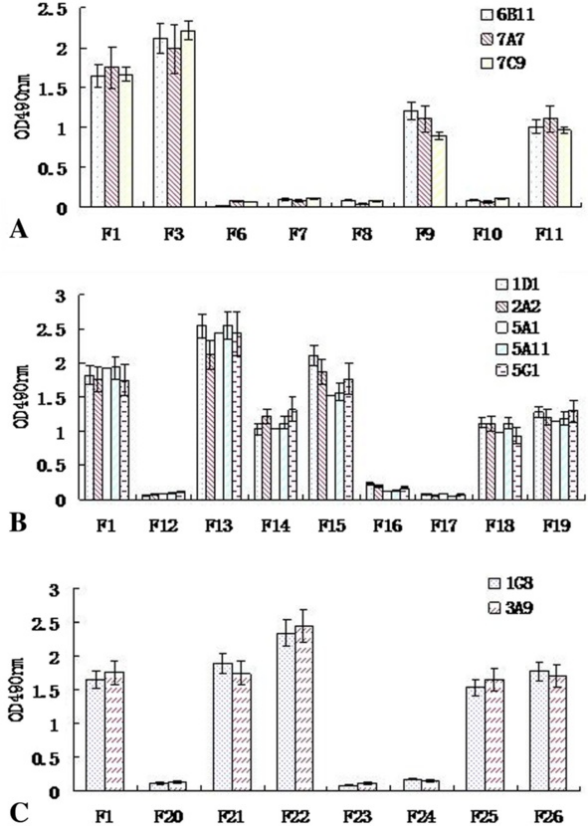
**Reactivity of McAbs with the different fragments of VP1 expressed in** ***E. coli*** **by ELISA. (A)** Reactivity of McAbs, 6E11, 7A7 and 7C9 with the fragments for identifying the epitope 2-7aa. **(B)** Reactivity of McAbs, 1D1, 2A2, 5A1, 5A11 and 5G1 with the fragments for identifying the epitope 19-25aa. **(C)** Reactivity of McAbs, 1G8 and 3A9 with the fragments for identifying the epitope 42-49aa.

Meanwhile, those purified truncated fragment proteins were used as the antigens in ELISA to detect the levels of those McAbs. The results showed that the OD_450_ values of McAbs (6E11, 7A7 and 7C9) in F1, F3, F9 and F11 groups were significantly higher than those in F6, F7, F8 and F10 (p < 0.01) (Figure 4A). The OD_450_ values of McAbs (1D1, 2A2, 5A1, 5A11 and 5G1) in F1, F13, F14, F15, F18 and F19 groups were markedly higher than those in F12, F16 and F17 groups (p < 0.05) (Figure 4B). The levels of McAbs (1G8 and 3A9) in the groups of F21, F22, F25 and F26 were notablely higher than those in F20, F23 and F24 (p < 0.01) (Figure 4C).

### Confirmation of the epitopes by using peptide-based ELISA

**Figure 5:**
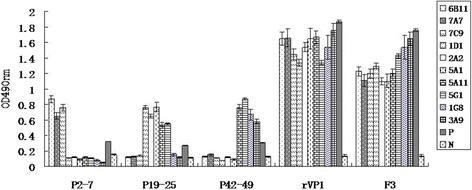
**Reactivity of the peptides with each McAbs and EMCV-specific serum.** The synthetic peptides, P2-7aa, P19-25aa and P42-49aa, were used as ELISA antigens. The recombinant VP1 and F3 fragment of VP1 expressed in *E.coli* was used as positive control. P: EMCV-specific serum; N: EMCV negative serum.

The synthetic peptides (P2-7aa, P19-25 and P42-49aa), purified rVP1 and GST-1-72aa (F3) proteins were used as ELISA antigens. The results showed that OD_450_ values of 6E11, 7A7 and 7C9 reacted with P2-7aa was highest in those McAbs. And the levels of McAbs (1D1, 2A2, 5A1, 5A11 and 5G1) reacted with P19-25aa were obviously higher than those of other McAbs (P < 0.05). McAbs 1G8 and 3A9 reacted with P42-49aa were significantly higher than other McAbs (P < 0.05). It indicated that the synthetic peptides P2-7aa, P19-25aa and P42-49aa have immune reactivity with the pepide specific McAbs, respectively (Figure 5).

However, the OD_450_ values of anti-EMCV mice serum were relatively lower than those of McAbs reacted with P2-7aa, P19-25aa and P42-49aa. It suggested that these synthetic peptides reacted weakly with EMCV-specific serum by peptide-based ELISA (Figure 5).

### Amino acid differences of the identified epitopes in different EMCV strains

**Figure 6:**
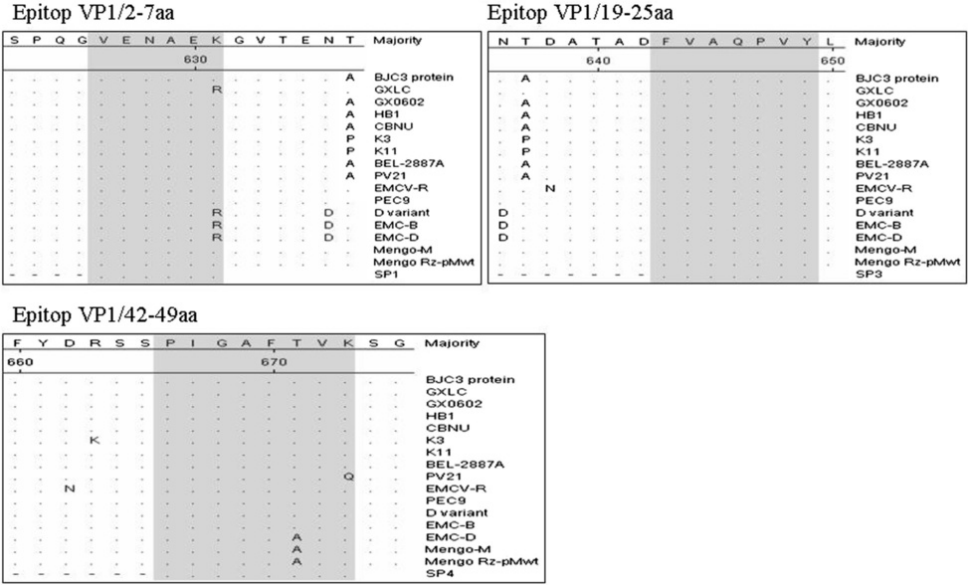
**Multiple alignments of the epitopes of VP1 among 16 EMCV isolates.** The amino acid sequences of the immunodominant epitopes identified were the gray area. Alignments of the amino acid sequences were made using DNAstar MegAlign software.

Alignment of the amino acid sequences of the different EMCV isolates revealed that the minimal epitope F(19)VAQPVY(25) was highly conserved among all the reference EMCV strains, the epitope V(2)ENAEK(7) was relatively conserved among all EMCV strains, except for a K7 → R7 change in GXLC, D variant, EMC-B and EMC-D strains. In addition, the epitope P(42)IGAFTVK(49) was conserved among the strains isolated from China. Whereas there were variable amino acids in different site of this region among the reference strains isolated from other countries (Figure 6).

### 3D model analysis of the three epitopes

**Figure 7:**
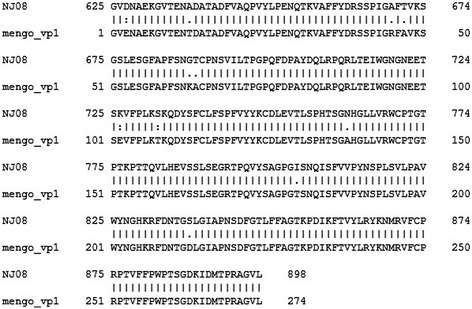
**Alignment of whole VP1 amino acid sequence of NJ08 with the selected strain mengo virus.** The black points represented different amino acids.

**Figure 8:**
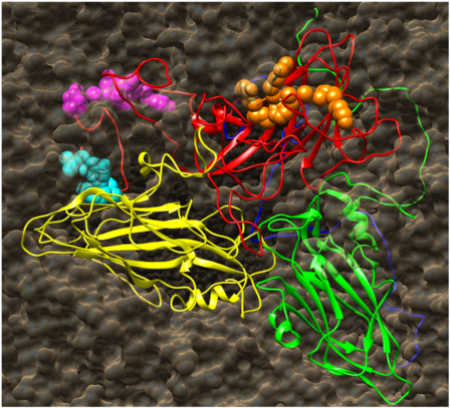
**The locations of the three epitopes.** The picture was about the complete particle of mengo virus. VP1 is shown in red ribbon, VP3 is shown in green ribbon, VP2 is shown in yellow ribbon, and VP4 is shown in blue ribbon. The 3 epitopes presented as spheres, V(2)ENAEK(7) is colored cyan, F(19)VAQPVY(25) is colored magenta, and P(42)IGAFTVK(49) is orange. The figure was generated using UCSF chimera [22]. (For interpretation of the references to color in this figure legend, the reader is referred to the web version of this article).

There existed a very close structural analog to NJ08 in 2 structures published in 1990 and 1994 with PDB accession numbers 2MEV and 1MEC respectively both for the mengo virus. The VP1 protein of NJ08 had very few amino acid changes and no deletions or additions in the sequence compared to the mengo virus VP1 sequence as shown in Figure 7. Therefore any of the 2 structures could be used a 3D model to map the epitopes within the context of the complete viral particle. As show in Figure 8, the 3 epitopes presented as spheres were shown within the context of the complete particle with colors of red for VP1, green for VP3, yellow for VP2 and blue for VP4. The epitopes V(2)ENAEK(7) and F(19)VAQPVY(25) shown as cyan and magenta spheres were partially buried in the viron. P(42)IGAFTVK(49) shown as orange spheres was located circa the 5-fold icosahedral axis of symmetry and was on the surface.

## Discussion

B cell epitopes are linear or conformational resulting from unique protein folding. They could be defined as regions on the surface of the native antigen that could bind to B-cell receptors or specific antibodies [21,23-25]. In this study, ten monoclonal antibodies (McAbs) against the VP1 of EMCV were prepared by inoculated with the purified killed EMCV, and then three minimal epitopes [V(2)ENAEK(7), F(19)VAQPVY(25), and P(42)IGAFTVK(49)] were identified in N terminus of the EMCV VP1, using these McAbs and a series of recombinant truncated VP1.

VP1 is one of the most important structures proteins of EMCV, which can stimulate the organism to produce neutralization antibody [18]. Immunization of mice with recombinant VP1 protein (plasmids or recombinant adenoviruses expressing small hairpin RNAs targeted to VP1 protein genes of EMCV), which could provide protective efficacy against EMCV [26,27]. DNA vaccines encoding an EMCV-D VP1 antigen confer protective immunity against heterologous challenge with EMCV-K, indicating that VP1 is a potential vaccine candidate for controlling EMCV infection [28]. Thus it is need to explore the epitopes related to the protection or diagnosis of this disease, even thought the residue at position 49, 62 and 100 are related to neutralization epitopes. In this study, the ten anti-VP1 McAbs all did not have the ability to neutralize the EMCV in cells cultures (data not shown here). However, they all could react with the EMCV antigen in Western blot and IFA and could be used for diagnosis of this virus.

Sequence analysis of both variants revealed one amino acid exchange within the capsid protein VP1, demonstrated that the diabetogenic and non-diabetogenic EMCV variants differing in only one single amino acid [29-32]. Studies of Theiler’s murine encephalomyelitis virus (TMEV) demonstrated that the amino acids on the loops exposed to the surface of the virion were important disease determinants [33]. The residue 49 was located in the loop connecting the βB and βC strands of VP1, which was exposed at the surface of the capsid and was known to be part of a neutralization epitope [34]. In this study, the results of alignment of the amino acid sequences of different EMCV isolates revealed that the epitope F(19)VAQPVY(25) was highly conserved among all the reference EMCV strains. There were only 1 or 2 amino acid residue different in the epitopes P(42)IGAFTVK(49) and V(2)ENAEK(7) between those strains. According to the model of mengo virus, the 3D model of VP1 of EMCV was also analyzed. In the picture, P(42)IGAFTVK(49) shown as orange spheres was located circa the 5-fold icosahedral axis of symmetry and was on the surface. Meanwhile, V(2)ENAEK(7) and F(19)VAQPVY(25) were at the N-terminus and located *under* the VP3 protein and sandwiched with the VP4 protein inside the particle. The obtain of the Mc-Abs against these two epitopes in this study might be explanted by the “breathing” of the N-terminal tail between the VP3 and VP2 proteins. These two epitopes could be used for diagnosis of this virus because of the results of alignment that these two regions were highly conserved among all the reference EMCV strains.

## Conclusions

Here, ten McAbs against EMCV VP1 were developed and three B-cells epitopes (2-7aa, 19-25aa and 42-49aa) were defined in VP1. The results may contribute to understand the antigenic structure of VP1 protein deeply and facilitate the development of diagnostic method of EMCV.

## Materials and methods

### Virus, cells, animals and other reagents

EMCV isolate NJ08 (GenBank Accession No. HM641897) used in this study was isolated and kept in our lab (Submitted 10-JUL-2010). BHK-21 cells were kept in Dulbecco’s Modified Eagle Medium supplemented with 10% fetal bovine serum. The SP2/0 cells were grown in RPMI 1640 Medium supplemented with 20% fetal bovine serum. Female BALB/c mice, 4–6 weeks of age, were purchased from the Laboratory Animal Centre of Nantong University. HAT and HT Media Supplement (50×) were purchased from Sigma. Horseradish peroxidase (HRP)-tagged goat anti-mouse IgG was purchased from Wuhan Boster Biological Technology Co. Rapid ELISA Mouse McAb Isotyping Kit was purchased from Pierce.

### Expression and purification of recombinant VP1 protein in E.coli and the purification of EMCV

The VP1 gene was amplified by PCR using the specific primers kept in our lab. PCR products were digested by *Bam*H I and *EcoR* I, and cloned into prokaryotic expression vector pET-28a(+) subsequently. The recombinant plasmids were verified by analysis and nucleotide sequencing, and then transformed into *E.coli* BL21 cells for fusion proteins expression. The transformants were cultured at 37°C in Luria-Bertani broth containing 0.05 mg/mL kanamycin until the optical density at 600 nm (OD_600_) reached 0.8. The cells were incubated for another 4 h in the presence of 1.0 mM isopropyl-β-D-thiogalactoside and harvested by centrifugation. The *E. coli*/pET-28a-VP1 transformants were lysed by sonication, and then centrifuged at 3000 g for 15 min at 4°C. The proteins were purified by ProBondTM Purification System, according to the manufacturer’s instructions (Invitrogen). The purified recombinant VP1 (rVP1) protein was detected by SDS-PAGE and Western blot, and the concentration was determined by NANODROP 2000 Spectrophotometer (Thermo).

EMCV was sedimented and purified from the culture medium with PEG-6000 by ultracentrifugation. The purified EMCV were analyzed by SDS-PAGE and Western blot with anti-EMCV sera.

### Development of monoclonal antibodies (McAbs)

The McAbs were developed and obtained as follows. Briefly, inactivated EMCV was combined with equal volumes of complete/incomplete Freund’s adjuvant (Sigma-Aldrich) at final concentration of 0.2 mg/ml. Each female BALB/c mice (6-week-old) was immunized with 0.1 mg emulsion at 3 weeks intervals for 9 weeks. Three days after the last injection, the spleens were surgically removed from the mice and fused with Mouse myeloma cells (SP2/0) using 50% (v/v) PEG. The fused hybridoma clones were screened by the indirect ELISA for McAbs that have strong reactivity with the rVP1 but not His protein or bound EMCV but not BHK-21 cells’ protein. Selected clones were subcloned by limiting dilution. Ascites fluids were induced in pristine-primed BALB/c mice. Finally, the McAbs were identified by Western blot and indirect immunofluorescence assay (IFA).

### Indirect ELISA

For screening the McAbs, ELISA plates were coated with the purified rVP1 protein and *E.coli*/pET-28a lysate (as the negative control) at the concentration of 0.002 mg/mL in buffer bicarbonate (pH9.6) at 4°C overnight or coated with the purified EMCV and BHK-21 cells’ protein at the concentration of 0.01 mg/mL as the same way above. After washing with PBS containing 0.05% Tween-80 (PBST) three times, the plates were blocked with 5% non-fat milk in PBST at 37°C for 2 h. Then wells were incubated with the supernatant of hybridoma at 37°C for 1 h. The sera isolated from immunized mice and the supernatant of SP2/0 cells were used as positive and negative controls, respectively. After three washes with PBST, wells were incubated with a 1:10,000 diluted goat anti-mouse IgG-HRP at 37°C for 1 h. After washing, the plates were incubated with substrate solution tetramethyl benzidine (TMB) at 37°C for 10 min, and the reaction was stopped with 2 M H_2_SO_4_ in each well (50 ml/well).The OD was read at 450 nm in an automatic ELISA plate reader. The criteria were judged as follows: the OD_450_ of the positive control should be more than 1.0, the OD_450_ of the negative controls should be lower than 0.2. The results were expressed as the ratio of OD_450_ produced by the samples compared to the negative control. Samples, giving a ratio value higher than 2.1, were considered to be positive.

For mapping the epitopes precisely, a series of truncated fragments of protein were expressed in *E.coli*. The epitopes recognized by McAbs were mapped by truncated VP1 fragments based on indirect ELISA as described above.

### Precise localization of epitopes

**Figure 9:**
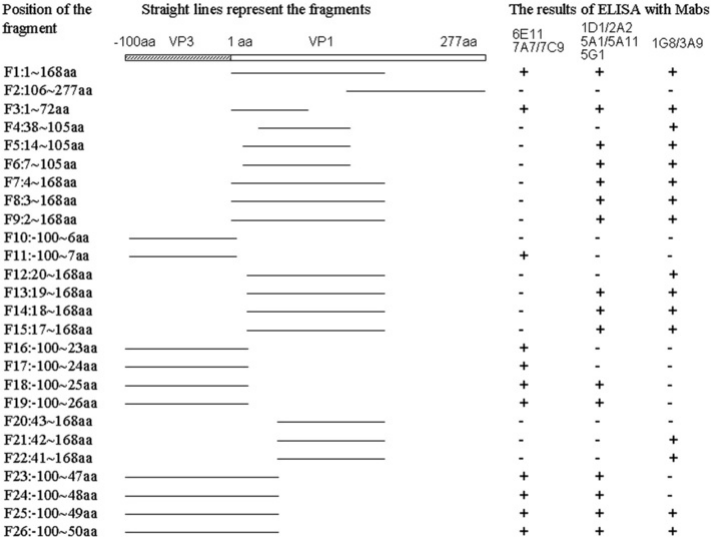
**Schematic diagram of the VP1 protein, showing the expression constructs of the VP1 fragments F1 to F26 and their interaction with McAbs in indirect ELISA analysis.** “+”: Positive. “-”: Negative.

In order to identify the minimal antigenic epitopes recognized by McAbs, the VP1 gene was divided into 26 truncated fragments (Figure 9). A series of primers (Table 1) were synthesized to amply the different truncated fragments of VP1 protein. PCR products were separately cloned into the *Xho* I and *Bam*H I sites of pGEX-6p-1 expression vector. The recombinant proteins were expressed in *E.coli* BL21 and identified by SDS-PAGE and Western blot with the GST-tagged monoclonal antibody (Boshide, Wuhan, China), and then were used to determine the epitopes recognized by McAbs using indirect ELISA and Western blot methods.

**Table 1:** **Primers set for amplification of the overlapping and truncated segments of VP1 gene**

**Fragment name**	**Primer sequence (5′-3′)**	**Fragment name**	**Primer sequence (5′-3′)**
F1:1 ~ 168aa	5′-GATGGATCCGGAGTAGAAAACGC-3′	F14:18 ~ 168aa	5′-AATGGATCCGACTTTGTGGCTCAACC-3′
	5′-TAACTCGAGCTAGCCTTCTGAGAG-3′		5′-ATTCTCGAGCTAGCCTTCTGAGAGGG-3′
F2:106 ~ 277aa	5′-CGCGGATCCCTTAAATCCAAACAG-3′	F15:17 ~ 168aa	5′-TAAGGATCCGCTGACTTTGTGGC-3′
	5′-GACCTCGAGTACTCTAGCATCAAG-3′		5′-ATTCTCGAGCTAGCCTTCTGAGAGGG-3′
F3:1 ~ 72aa	5′-GATGGATCCGGAGTAGAAAACGC-3′	F16:-100 ~ 23aa	5′-TTAGGATCCGGAGCGGGCAAG-3′
	5′-CACCTCGAGCTAGTCAGTATCACTG-3′		5′-ATCCTCGAGCTATGGTTGAGCCACAAA-3′
F4:38 ~ 105aa	5′-TAAGGATCCGATAGGTCCAGTC-3′	F17:-100 ~ 24aa	5′-TTAGGATCCGGAGCGGGCAAG-3′
	5′-GACCTCGAGCTACGGAAAGACTTTTG-3′		5′-CATCTCGAGCTAAACTGGTTGAGCCA-3′
F5:14 ~ 105aa	5′-ATTGGATCCGACGCAACTGCTGA-3′	F18:-100 ~ 25aa	5′-TTAGGATCCGGAGCGGGCAAG-3′
	5′-GACCTCGAGCTACGGAAAGACTTTTG-3′		5′-CATCTCGAGCTAGTAAACTGGTTGAGCC-3′
F6:7 ~ 105aa	5′-CAGGGATCCAAAGGGGTCACTGAAAAC-3′	F19:-100 ~ 26aa	5′-TTAGGATCCGGAGCGGGCAAG-3′
	5′-GACCTCGAGCTACGGAAAGACTTTTG-3′		5′-AGCCTCGAGCTACAAGTAAACTGGTTGA-3′
F7:4 ~ 168aa	5′-GCAGGATCCAACGCTGAAAAAG-3′	F20:43 ~ 168aa	5′-TAAGGATCCATTGGTGCCTTCACCG-3′
	5′-ATTCTCGAGCTAGCCTTCTGAGAGGG-3′		5′-ATTCTCGAGCTAGCCTTCTGAGAGGG-3′
F8:3 ~ 168aa	5′-CCGGGATCCGAAAACGCTGAAAAA-3′	F21:42 ~ 168aa	5′-ATTGGATCCCCCATTGGTGCCTTC-3′
	5′-ATTCTCGAGCTAGCCTTCTGAGAGGG-3′		5′-ATTCTCGAGCTAGCCTTCTGAGAGGG-3′
F9:2 ~ 168aa	5′-CGAGGATCCGTAGAAAACGCTGAA-3′	F22:41 ~ 168aa	5′-ATAGGATCCAGTCCCATTGGTGCC-3′
	5′-ATTCTCGAGCTAGCCTTCTGAGAGGG-3′		5′-ATTCTCGAGCTAGCCTTCTGAGAGGG-3′
F10:-100 ~ 6aa	5′-TTAGGATCCGGAGCGGGCAAG-3′	F23:-100 ~ 47aa	5′-TTAGGATCCGGAGCGGGCAAG-3′
	5′-GCACTCGAGCTATTCAGCGTTTTCTACT-3′		5′-TTCCTCGAGCTAGGTGAAGGCACCAAT-3′
F11:-100 ~ 7aa	5′-TTAGGATCCGGAGCGGGCAAG-3′	F24:-100 ~ 48aa	5′-TTAGGATCCGGAGCGGGCAAG-3′
	5′-GGCCTCGAGCTATTTTTCAGCGTTTTCTAC-3′		5′-ATACTCGAGCTACACGGTGAAGGCACC-3′
F12:20 ~ 168aa	5′-TCAGGATCCGTGGCTCAACCAGTTTAC-3′	F25:-100 ~ 49aa	5′-TTAGGATCCGGAGCGGGCAAG-3′
	5′-ATTCTCGAGCTAGCCTTCTGAGAGGG-3′		5′-ATACTCGAGCTACTTCACGGTGAAGGC-3′
F13:19 ~ 168aa	5′-ACTGGATCCTTTGTGGCTCAACCAG-3′	F26:-100 ~ 50aa	5′-TTAGGATCCGGAGCGGGCAAG-3′
	5′-ATTCTCGAGCTAGCCTTCTGAGAGGG-3′		5′-AACCTCGAGCTAGGACTTCACGGTGAA-3′

### Western blot

To identify the proteins expressed in *E. coli* (VP1 and truncated VP1 proteins), the proteins were separated by SDS-PAGE (5-12%) and transferred to nitrocellulose membrane (Pall Corporation). The McAbs (dilution of 1:10–1:50) bound to the proteins on the membrane was detected by goat anti-mouse IgG-HRP (Boshide, Wuhan, China) at 37°C for 1 h. Detection was performed using chemiluminescenceluminol reagents (SuperSignal West Pico Trial kit, PIERCE).

### Indirect immunofluorescence assay (IFA)

BHK21 cells infected with EMCV NJ08 strain in 96-well culture plate were rinsed with PBS and fixed with cold ethanol for 45 min at 4°C. The cells were washed, and then incubated with the McAbs (1:10 diluted in PBST) for 1 h at 37°C. After washing with PBST, the cells were incubated with goat anti-mouse IgG conjugated with fluorescein (1:50 diluted in PBST) for 30 min at 37°C. After rinsing by five times, cells were kept in PBS and observed under a ZEISS fluorescence microscope.

### Peptide-based ELISA

Three linear epitopes (P2-7aa, P19-25aa and 42-49aa) identified in this study were synthesized using Fmoc solid-phase chemistry (Invitrogen) with >98% purity. They were coated in Reacti-Bind™ Amine-binding, Maleic Anhydride 96-well plates (Pierce, U.S.A.) at the concentration of 10ug/ml for 5 h at room temperature. Meanwhile, purified GST-1-72aa (F3) and rVP1 protein were used as positive antigens control. Mice anti-EMCV serum (at dilution of 1:100) were added as the primary antibody and incubated at 37°C for 1 h. The SPF mice serum was used as negative antibody control. The following detection steps were the same as described above.

### Alignment of VP1 epitopes sequences

Nucleotide sequences of EMCV strains from different countries were retrieved from Genbank. The amino acid sequences of identified B-cell epitopes were aligned using DNAstar MegAligen software. The representative EMCV strains were listed in Table 2.

**Table 2:** **The EMCV reference strains cited in this study**

**Virus designation**	**GenBank accession no**	**Geographic origin**	**Species**	**Clinical field observation**
GXLC	FJ897755	China	Swine	Myocarditis
BJC3	DQ464062	China	Swine	Reproductive failure
HB1	DQ464063	China	Swine	Myocarditis
K3	EU780148	Korea	Swine	Reproductive failure
K11	EU780149	Korea	Swine	Reproductive failure
CBNU	DQ517424	Korea	Swine	Reproductive failure
BEL-2887A	AF356822	Belgium	Swine	Reproductive failure
PV21	X74312	Germany	Swine	Myocarditis
D variant	M37588	USA	Swine	Myocarditis
EMC-B	M22457	USA	Swine	Myocarditis
EMC-D	M22458	USA	Swine	Myocarditis
GX0602	FJ604853	China	Mice	Myocarditis
Mengo-M	L22089	Uganda	Monkey	Myocarditis
Mengo Rz-pMwt	DQ294633	USA		Myocarditis
EMCV-R	M81861	USA	Chimpanzee	Myocarditis
PEC9	DQ288856	USA	Mice	Myocarditis

### 3D model analysis of these epitopes

Molecular graphics and analyses were performed with the UCSF Chimera package. Chimera is developed by the Resource for Biocomputing, Visualization, and Informatics at the University of California, San Francisco (supported by NIGMS P41-GM103311).

### Statistics

The differences in the level of antibodies between different groups were determined by One-way repeated measurement ANOVA and Least significance difference (LSD). Differences were considered statistically significant when p < 0.05.

## Abbreviations

McAbs: Monoclonal antibodies
ORF: Open reading frame
